# Tyrosine Kinase Inhibitors Play an Antiviral Action in Patients Affected by Chronic Myeloid Leukemia: A Possible Model Supporting Their Use in the Fight Against SARS-CoV-2

**DOI:** 10.3389/fonc.2020.01428

**Published:** 2020-09-02

**Authors:** Sara Galimberti, Mario Petrini, Claudia Baratè, Federica Ricci, Serena Balducci, Susanna Grassi, Francesca Guerrini, Elena Ciabatti, Sandra Mechelli, Antonello Di Paolo, Chiara Baldini, Laura Baglietto, Lisa Macera, Pietro Giorgio Spezia, Fabrizio Maggi

**Affiliations:** ^1^Department of Clinical and Experimental Medicine, University of Pisa, Pisa, Italy; ^2^Hematology, AOUP, Pisa, Italy; ^3^Department of Translational Research and New Technologies in Medicine and Surgery, University of Pisa, Pisa, Italy

**Keywords:** CML, TKIs, imatinib, nilotinib, TTV, immunity, NanoString, COVID-19

## Abstract

SARS-CoV-2 is the viral agent responsible for the pandemic that in the first months of 2020 caused about 400,000 deaths. Among compounds proposed to fight the SARS-CoV-2-related disease (COVID-19), tyrosine kinase inhibitors (TKIs), already effective in Philadelphia-positive acute lymphoblastic leukemia (Ph+ ALL) and chronic myeloid leukemia (CML), have been proposed on the basis of their antiviral action already demonstrated against SARS-CoV-1. Very few cases of COVID-19 have been reported in Ph+ ALL and in CML Italian cohorts; authors suggested that this low rate of infections might depend on the use of TKIs, but the biological causes of this phenomenon remain unknown. In this study, the CML model was used to test if TKIs would sustain or not the viral replication and if they could damage patient immunity. Firstly, the infection and replication rate of torquetenovirus (TTV), whose load is inversely proportional to the host immunological control, have been measured in CML patients receiving nilotinib. A very low percentage of subjects were infected at baseline, and TTV did not replicate or at least showed a low replication rate during the follow-up, with a mean load comparable to the measured one in healthy subjects. Then, after gene expression profiling experiments, we found that several “antiviral” genes, such as *CD28* and *IFN gamma*, were upregulated, while genes with “proviral” action, such as *ARG-1, CEACAM1*, and *FUT4*, were less expressed during treatment with imatinib, thus demonstrating that TKIs are not detrimental from the immunological point of view. To sum up, our data could offer some biological explanations to the low COVID-19 occurrence in Ph+ ALL and CML patients and sustain the use of TKIs in COVID-19, as already proposed by several international ongoing studies.

## Introduction

In March 2020, the pandemic sustained by the Severe Acute Respiratory Syndrome-Coronavirus 2 (SARS-CoV-2) was officially declared, involving the entire world, after which a new Coronavirus was isolated for the first time in China, in the Hubei province, in December 2019. On June 7, 2020, 6,750,521 cases (USA: 1,886,794, Europe: 2,271,919, and China: 84,629) and 395,779 deaths (USA: 100,038, Europe: 182,790, and China: 4,645) had been recorded (https://www.cdc.gov/coronavirus/2019-nCoV/index.html), an enormous number that well-reflects the gravity of the worldwide situation.

The disease caused by SARS-CoV-2, now known as Coronavirus Disease 19 (COVID-19), is characterized by different clinical manifestations and severity, ranging from cough and cold across fever and mild symptoms to capillary leak, respiratory distress, thrombotic events, and renal, hepatic, and coagulation failure (1). Recently, many efforts have been made in order to detect, isolate, and characterize the virus; understand the COVID-19 pathogenesis; and, especially, find effective treatments.

The SARS-CoV-2 structure was rapidly characterized: it is an RNA virus, with a nucleotide identity higher than 80% with the genome of SARS-CoV-1, the virus responsible for the Asian outbreak of 2002/2003 (2). One of the viral proteins, the surface spike (S) protein, is fundamental for virus attachment, fusion, and entry into the human host cells through the angiotensin-converting enzyme 2 (ACE2) receptor (3). More recently, the dipeptidyl peptidase 4 (DPP4) (also known as CD26) (4) has been characterized as a further virus receptor. DPP4 axis is used by virus for blocking autophagy, for impairing host immune response and sustaining the hyper-inflammatory status (5). Interestingly, both angiotensin and CD26 are involved in the senescence phenomenon, which, in addition to being responsible for further virus dissemination, might explain why COVID-19 is more severe in older people and why azithromycin seems to be effective (6). Coronavirus enters the human cells via a pH- and receptor-dependent way (7); once it has entered the host cells, the virus uses the proteasome ubiquitin system to destroy the host antiviral proteins and increase the production of those proteins that are necessary for its replication (8).

The COVID-19 pathogenesis has been ascribed to the “cytokine storm” that is characterized by increased levels of IL1, IL6, IFN gamma, TNF alpha, IL12, and IL15, in a way similar to the one observed in the macrophage activation syndrome (9), in the cytokine release disease following CAR-T infusion (10), and in the acute graft-versus-host disease (aGVHD), a complication of allogeneic stem cell transplantation (11).

Among the available therapeutic approaches against COVID-19, some of them belong to the rheumatological and hematological armamentarium (12). The efficacy of chloroquine and hydroxychloroquine, which act as immunomodulating agents by modifying the pH of endocytic vesicles (so blocking the first infection phases), is a matter of debate. Indeed, several studies focused their attention on the increased risk of prolongation of QTc and possible onset of arrhythmias that these compounds, especially in combination with antibiotics, seem to induce (13). On the other hand, a meta-analysis conducted on nine different studies sustained the efficacy of these drugs (14). Recently, Lancet retracted a paper published 13 days before where the authors neglected the efficacy of chloroquine and hydroxychloroquine, used alone or in combination with macrolides, in a series of 14,888 patients and 81,144 untreated controls (15), once again making this issue a matter of debate. Other drugs borrowed by the “rheumatological world” have been used in COVID-19 because of their anti-inflammatory power: tocilizumab, an anti-IL6 antibody; anakinra, an anti-IL1 antibody; and baricitinib, an anti-JAK1 compound (16).

Moreover, other possible effective drugs have been derived from the “hematological world,” such as ruxolitinib, a powerful JAK1/2 inhibitor, already effective also in aGVHD (17, 18); begelomab, an anti-CD26 monoclonal antibody that can block the viral occupation of CD26 receptors, restore autophagy, and reduce the hyper-inflammatory status (5); and proteasome inhibitors that block the synthesis of the proteins necessary for the production of new virions (8).

In March 31 2020, a randomized clinical trial, “COUNTER-COVID—Oral imatinib to prevent pulmonary vascular leak in COVID-19,” was registered in Netherlands (https://www.clinicaltrialsregister.eu/ctr-search/trial/2020-001236-10/NL). The primary endpoint of this study was to test whether imatinib might reduce the aggressiveness of COVID-19 pneumonitis. Other clinical trials involving imatinib, alone (NCT04357613) or in combination with baricitinib, lopinavir/ritonavir (NCT04346147), or hydroxychloroquine (NCT04356495), were registered on the “ClinicalTrials.gov” website and are now ongoing.

The basis for the possible efficacy of imatinib in COVID-19 can be determined from previous studies conducted in the SARS-CoV-1 model, where imatinib reduced the number of infected cells by preventing syncytia formation induced by the viral S protein. Considering that Abl1 kinase controls the cytoskeletal rearrangement, the authors suggested that imatinib, inhibiting Abl1, could interfere with the actin dynamics required for virus entry and cell fusion (19, 20). Two recently published Italian surveys, one focused on patients affected by Philadelphia-positive acute lymphoblastic leukemia (Ph+ ALL) and the other one on chronic myeloid leukemia (CML) cases can be the real proof that these observations done *in vitro* are true also *in vivo*. Indeed, among 267 adults with Ph+ ALL currently managed in participating centers (including 128 subjects coming from the four Italian regions most affected by the new coronavirus), only one patient has proven to be COVID-19 positive, with a quite rapid resolution of pneumonitis and cellulitis (21). The second article (22) reports that in 6,883 CML patients observed at 51 centers, only 12 cases were SARS-CoV-2 positive. The authors of these articles hypothesized that this low percentage of infections and absence of COVID-19-related severe symptoms might be related to a “protective” action exerted by tyrosine kinase inhibitors (TKIs).

With these assumptions, we conducted two different types of experiments, with the aim of trying to explain why TKIs would protect Ph+ ALL and CML patients from COVID-19.

Firstly, as a “viral” model, we employed the torquetenovirus (TTV), a DNA-virus assigned to the family of Anelloviridae (23), detectable in more than 60% of the asymptomatic healthy subjects (24). It has been reported that TTV load significantly increases after immunosuppressive therapy and solid organ or stem cell transplantation. Translated in the COVID-19 context, if TKIs would sustain the coronavirus infection or replication, we might expect to observe a significant increase of TTV load during treatment of our patients with nilotinib.

Then, we analyzed the immune profile of five CML patients during imatinib treatment in order to test if and how much several hundreds of immunity-related genes were modulated by this TKI. Indeed, we previously reported about the anti-inflammatory power of imatinib (25), which, when translated in the COVID-19 scenario, might represent an important strong point. Nevertheless, we could assume that this anti-inflammatory action could be accompanied by an immune control level reduction, which might be detrimental for the final fight of the virus. With these premises, in the second part of the study, we evaluated the middle-term activity of imatinib on the immune system of our CML patients.

## Patients and Methods

### Patients

#### Nilotinib Cohort

TTV load was measured by quantitative real-time PCR in 60 peripheral blood samples from 10 CML patients in the chronic phase receiving nilotinib as a first-line therapy. The clinical features and outcomes of these subjects are reported in Table 1. In particular, six patients were male and four female; their median age was 46 years; the Sokal risk score was high in three cases, intermediate in six, and low in one. All except one achieved an early molecular response (*BCR-ABL1/ABL1* ratio ≤ 10% IS after 3 months of treatment), and 9/10 achieved MR3 (*BCR-ABL1/ABL1* ratio ≤ 0.1% IS) at 12 months.

**Table 1:** Features of patients treated with nilotinib as first-line treatment tested for TTV load.

**Feature**	**Number (%)**
Total patients number (nilotinib)	10
**Sex**	
Male	6 (60%)
Female	4 (40%)
Age, median (range)	46 (27–63)
**Sokal Risk**	
Low	3 (30%)
Intermediate	6 (60%)
High	1 (10%)
EMR (BCR-ABL1/ABL1 ≤ 10% at 3 months)	9 (90%)
CCyR (no Philadelphia) at 6 months	9 (90%)
MR3 at 12 months (BCR-ABL1/ABL1 ≤ 0.1%)	9 (90%)

#### Imatinib Cohort

A possible deregulation of 770 mRNAs caused by imatinib was measured by NanoString technology in peripheral blood samples of five CML patients in the chronic phase after 6 months of 400 mg/day of this TKI with respect to the diagnosis. Clinical features and outcomes of these subjects are reported in Table 2. In particular, three patients were male and two female; their median age was 55 years; the Sokal risk score was high in one case, intermediate in three, and low in another one. In this cohort, two patients were resistant to imatinib, while the other three became optimal responders, according to the ELN 2013 guidelines (26). Before participating in the study, all patients signed an informed consent approved by the Ethical Committee of the AOUP where they declared to donate leftover samples used for diagnostics for further scientific non-profit purposes.

**Table 2:** Features of patients treated with imatinib as first-line treatment enrolled in the gene expression profile assays.

**Feature**	**Number (%)**
Total patients number (imatinib)	5
**Sex**	
Male	3 (60%)
Female	2 (40%)
Age, median (range)	55 (55–72)
**Sokal Risk**	
Low	1 (20%)
Intermediate	3 (60%)
High	1 (20%)
EMR (BCR-ABL1/ABL1 ≤ 10% at 3 months)	3 (60%)
CCyR (no Philadelphia) at 6 months	3 (60%)
MR3 at 12 months (BCR-ABL1/ABL1 ≤ 0.1%)	3 (60%)

### Methods

#### TTV Load Measure

Viral DNA was extracted from 200 μl whole peripheral blood anticoagulated with EDTA using QIAamp DNA minikit (Qiagen, Chatsworth, CA, USA) and stored at −20°C. Presence and load of TTV genome were determined by single-step quantitative real-time PCR, as described elsewhere (27). The method showed high sensitivity, being able to identify as positive samples those containing ≥10 viral genomes per milliliter; the specificity had been previously demonstrated, since this technique does not detect other human anelloviruses.

#### BCR-ABL1/ABL1 Ratio IS Detection

The *BCR-ABL1/ABL1* ratio was measured by quantitative PCR on the concomitantly harvested peripheral blood, according to the standardized operative procedures of the Italian cooperative group GIMEMA LabNet (www.gimema.it/labnet-cml/). A minimum of 20,000 *ABL1* copies was necessary for considering a sample as “evaluable”; 32,000 *ABL1* copies were necessary for defining MR4.5 or 100,000 *ABL1* copies for MR5, according to the ELN guidelines (28).

#### NanoString Assays

NanoString technology (NanoString, Seattle, USA) has been employed for analyzing the immune transcriptome profile of five CML patients after 6 months of treatment with imatinib in comparison with diagnosis. The “Human nCounter Myeloid Innate Immunity panel” that measures the expression of 770 genes involved in 19 different pathways, fundamental for the innate immune response, has been adopted.

### Statistical Analysis

Results from the NanoString were analyzed by the nCounter Advanced Analysis 2.0 software that allows us to identify genes significantly upregulated or downregulated, design volcano plots, and perform principal component analysis. For the remaining data, SPSS software version 22 (IBM, Bologna, Italy) was used. Viral load variable was used after transformation of TTV load in log format. Fisher's exact test has been applied to the contingency tables. Differences between distributions were calculated by a non-parametric Mann–Whitney *U* test. The association among variables was evaluated by the Kruskal–Wallis test. Correlations between variables were assessed using the Spearman *r* correlation coefficient and Student's *t*-test. Regression analyses were conducted to evaluate the association between the dependent variable TTV viremia and *BCR-ABL1/ABL1* ratio. All *p*-values presented are based on two-tailed tests, and *p* ≤ 0.05 was considered as statistically significant.

## Results

### TTV Infection and Replication Rates in CML Patients

TTV load was measured by quantitative real-time PCR in 60 peripheral blood samples from 10 CML patients in the chronic phase receiving nilotinib 600 mg/day as a first-line therapy. At diagnosis, only two patients showed detectable TTV genome; both TTV-positive patients were male, aged 40 and 53 years; no correlation with any of their clinical features and TTV presence was found. Because a correlation between age and sex with TTV replication has been previously reported in healthy subjects (29), we tested if this observation would be reproducible also in our series. Even if our cohort was very small, no correlation between age or sex and TTV infection rate was found.

Then, we analyzed the TTV load at different time points in order to test if in the TTV-negative cases, the virus started to replicate during treatment with nilotinib and if and how TTV load eventually would change during follow-up. Eleven patients were tested after 3, 6, and 9 months; 9 cases were also tested at 12 months, 15 at 18 months, and 3 patients also after 21 months of therapy.

Overall, during follow-up, 41 samples (68%) were TTV negative, while 19 (32%) presented a TTV quantifiable load. The mean TTV load in positive cases was 2.8 log copies per milliliter (95% confidence interval: 2.5–3.1 log copies per milliliter). It is worth remembering that in different series of immunocompromised patients, the mean TTV load was 4.1 log copies per milliliter (95% confidence interval: 3.9–4.3 log copies per milliliter) (25, 26, 29–32) and that the mean TTV load in healthy blood donors was 2.3 log copies per milliliter (95% confidence interval: 1.7–2.9 log copies per milliliter) (24).

When TTV load was measured at the last time points of follow-up, 9 of the 10 treated CML patients showed either unchanged or slightly increased values (<0.4 log copies per milliliter of variation) relative to baseline. In particular, in one of the two initially TTV-positive patients, TTV did not replicate already at the first time point (after 3 months of therapy), while in the second case, the mean value of TTV load measured at six different time points was the same as that measured baseline (2.7 log copies per milliliter). In the subgroup of cases who were initially TTV negative, four out of eight remained TTV negative during the whole follow-up; in the other four, TTV load was detectable in half of the occasions, but with a maximum increase value of 0.4 log copies per milliliter. When we analyzed TTV load with respect to molecular response assessed by the *BCR-ABL1/ABL1* ratio measured by real-time PCR at the same time point, a statistically significant correlation between genome TTV detection and absence of optimal response was found. Indeed, according to the ELN 2013 classification (28), we counted seven failing time points: in all these occasions, the TTV genome was detectable. On the other hand, we had 53 time points where molecular response was optimal or a warning: in 33 of these occasions, TTV was not detectable (*p* = 0.02).

In conclusion, this first part of the study showed that nilotinib does not sustain the viral replication, even with a medium-term follow-up.

### Deregulation of Immunity-Related Genes in CML Patients

In the second phase of our study, we employed the NanoString technology for analyzing the expression of 770 inflammation- and immunity-related genes in five CML patients before and after 6 months of treatment with imatinib, with the aim of testing the impact of this TKI on the possible immunological control of viral infection.

Overall, 58 genes were deregulated by imatinib, with 18 genes being upregulated and 40 downregulated. We previously reported that some genes involved in several different autoimmune/inflammatory conditions were downregulated by imatinib (25), which might have a positive impact on COVID-19. Interestingly, 20 out of these 58 deregulated genes were strictly correlated with immune or antiviral response, so representing the focus of the present study (see Supplemental File 1 for the original raw data).

Among these 20 genes, 11 appeared upregulated and 9 downregulated during treatment with imatinib (see Table 3). In more detail, we found a reduced expression of *Arginase 1* (*ARG-1*) (44), *Complement C3a Receptor 1* (*C3AR1*) (46), *Carcinoembryonic antigen-related cell adhesion molecule-1* (*CEACAM1*) (45), *Gelsolin* (*GSN*) (49), *Nectin 1* (50), and *Fucosyltransferase 4* (*FUT4*) (48), all genes that usually play a “proviral” role. Indeed, *ARG-1* displays a negative effect on immunity in several contexts, including hematological neoplasms: by depleting the microenvironment of arginine, which is essential for T-lymphocyte function, arginase makes them anergic. In CML, *ARG-1* has been shown to be highly expressed at diagnosis, when myeloid-derived suppressor cells are very active against T-cell activity (53). *CEACAM1* is an important regulator of virus-specific CD8+ T-cell functions. In an *in vitro* model, treatment with anti-CEACAM1 antibody prevented CD8+ T-cell exhaustion, thus improving the control of viral infections (54). *FUT4* is a gene strictly involved in the PD1 axis, whose overexpression has been associated with a shorter survival in lung adenocarcinoma (48). Consequently, *FUT4* downregulation could contribute to the inhibition of the PD1–PDL1 axis, with consequent recovery of the immune disease control.

**Table 3:** In the table are listed genes that were upregulated (in red) or downregulated (in green) in CML patients during treatment with imatinib.

**GENE ID**	**Function**	**Final effect**	**References**
CCL5	Activates NK	Anti-infective	(33)
CCR5	Activates NK	Anti-infective	(34)
CD28	Low in severe COVID-19	Anti-infective	(35)
CD74	Blocks macrophage activation	Pro-infective	(36)
CX3CR1	High in antifungal resp	Anti-infective	(37)
CXCL16	High in antiviral resp	Anti-infective	(38)
CXCR3	High in T effector	Anti-infective	(39)
HAVCR2	NK mature marker	Anti-infective	(40)
IFNG	Antiviral	Anti-infective	(41)
NFATC2	Increases T cells	Anti-infective	(42)
TLR3	Antiviral	Anti-infective	(43)
ARG1	Immunosuppressive	Pro immune	(44)
CEACAM1	Inhibits T lynf	Pro-infective	(45)
C3AR1	Neutrophil chemotaxis antagonist	Anti-infective	(46)
COL17A1	Induces IL7 that sustains T and B lynf	Anti-inflammation	(47)
FUT4	Increases bacterial infections	Anti-infective	(48)
GSN	Increases NK apoptosis	Anti-infective	(49)
NECTIN1	High in chlamydial infection	Anti-infective	(50)
RNASE2	Antiviral	Pro-infective	(51)
RNASE3	Antiviral	Pro-infective	(52)

*For each gene, the respective functions and the final effect after deregulation done by this TKI are indicated. In violet are the effects that could sustain a possible virus replication*.

On the other hand, imatinib upregulated some genes that could finally exert an “antiviral” action by sustaining the host immunological control of the viral attack, such as *CD28* (35), *Interferon gamma* (*IFN gamma*) (41), *C-C Motif Chemokine Ligand 5* (*CCL5*) (33), *C-C chemokine receptor type 5* (*CCR5*) (34), and *Toll-like receptor 3* (*TLR3*) (43). Particularly interesting is *CD28*, a co-stimulatory molecule also used for CAR-T production (55). *CD28* is located immediately downstream of *PD1*, thus participating in the PD1-derived T-cell suppression (56). The role of CD28 in COVID-19 has been recently investigated: its expression on CD8+ T cells seems to be lower in patients with severe relative to those with mild COVID-19 (57); consequently, its overexpression induced by imatinib might be not detrimental. CCL5 is another interesting chemokine with antiviral action: indeed, in a murine model, *CCL5*-overexpressing NK cells were hyperactivated and made animals resistant to the viral infection (33). The pro-immune action of *IFN* gamma is well-known (41); in CML, interferon has a long history of successes, and it seems to be able to delete *ABL1* mutations when added to TKIs, hence making patients sensitive again to treatment (58). Finally, also, the increased expression of *TLR3* might be positively translated into the COVID-19 context: indeed, it has been previously reported that its overexpression protects neutropenic mice from meningoencephalitis (59).

**Figure 1:**
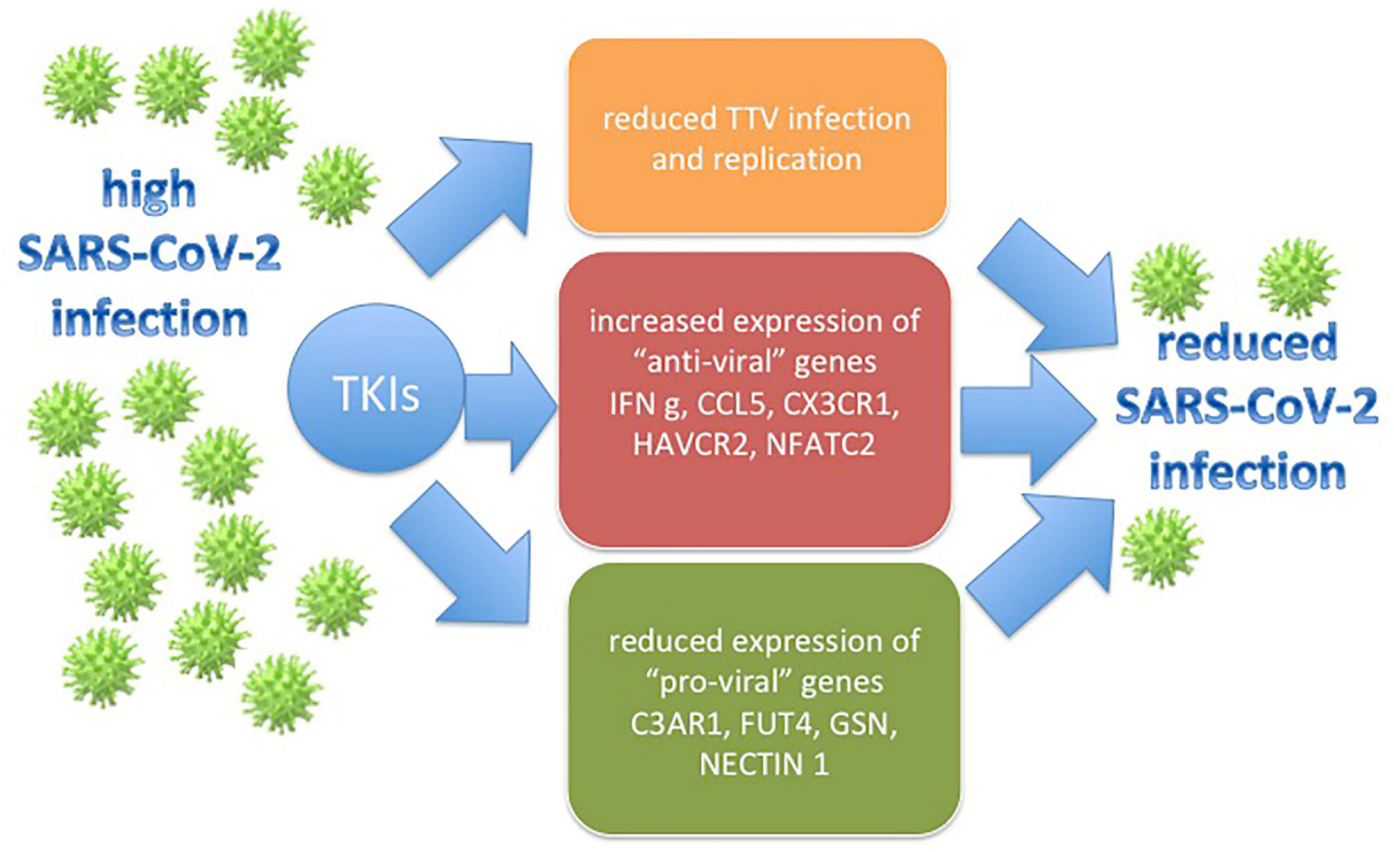
The figure represents how the effects that TKIs have on the CML models employed in the study (the TTV replication and immune gene deregulation) might be translated in the SARS-CoV-2 context, with a possible final antiviral action.

In conclusion, even if performed on a small series of cases, results from our experiments might support the idea that imatinib could sustain the immunological control of infected subjects (Figure 1). These findings, translated in the coronavirus scenario, could help to explain why very few patients affected by Ph+ ALL and CML developed COVID-19 symptoms.

## Discussion

The SARS-CoV-2 outbreak and its related disease, COVID-19, represent a new world war that in just a few months produced about 400,000 deaths. Consequently, all of the scientific community is now working to find effective diagnostic tests and therapeutic approaches. Recently, a Dutch group started to treat COVID-19 pneumonitis with imatinib, a TKI already employed in CML (60), Ph+ ALL (61), and gastrointestinal stromal tumor (GIST) (62). The hypothesis that imatinib could be effective also in COVID-19 was based on some previous experimental observations done in SARS and MERS models where imatinib was able to block the virus entrance into human host cells (20). Recently, very few cases of COVID-19 have been reported in the Italian cohort of Ph+ ALL and CML patients (21). Considering that it has been proven that at diagnosis, the immunity of these patients is severely impaired (63), the low infection rate observed during the 2020 pandemic could prove that TKIs play an antiviral role or, at least, could not impair the host response against the new coronavirus.

In our study, we tested if nilotinib could favor or not the viral replication by using the TTV model. We found that in 50% of patients, TTV did not replicate at all evaluated time points and that, in subjects where the TTV load was measurable, its replication rate was low. Indeed, the mean TTV load in positive cases was 2.8 log copies per milliliter, lower than that observed in immunocompromised patients (where the mean TTV load was 4.1 log copies per milliliter) (25, 26, 29–32) and comparable with that of healthy individuals (where the mean TTV load was 2.3 log copies per milliliter) (24). In our opinion, these results might be considered as a good proof that nilotinib does not favor the viral replication.

Moreover, in our series, we observed that only 20% of patients presented a measurable TTV load baseline, a result that mimics the very low rate of infection by the new coronavirus in Ph+ ALL and CML patients. The mechanisms that TTV uses for entering the host cells are still unknown, and we might hypothesize that TTV receptors on CML cells could be lower or absent. This may not be relevant in the coronavirus scenario, because we know that CD26, highly expressed in CML leukemic cells (64), is one of the SARS-CoV-2 receptors. Consequently, in our opinion, the very low percentage of CML patients found to be SARS-CoV-2 positive could be not explained by the inability of the virus to attack the host cells. On the contrary, we can consider that, as already described for SARS-CoV-1, Bcr-Abl1 tyrosine kinase, expressed at very high levels at diagnosis, could change the conformational status of the host cells, making them “impenetrable” by the virus. Indeed, Bcr-Abl1 tyrosine kinase modifies the actin structure and cytoskeleton function (65), probably also by actin adaptors, such as FAK, whose codifying gene is expressed at a very low level in CML, especially in advanced phases of the disease (66). In conclusion, we cannot exclude that Bcr-Abl1 might make Ph+ cells more impenetrable for viruses compared with normal cells. Moreover, about the low rate of TTV replication during treatment with nilotinib, we can hypothesize that some “antiviral” genes could be more expressed. In our previous study, we observed that imatinib upregulated two “antiviral” genes: *Interferon-stimulated gene 15* (*ISG15*) and Mov10 RISC Complex RNA Helicase (*MOV10*) (67). *ISG15* has been reported to exert a direct negative effect on viral replication, probably by restoring autophagy and sustaining NK proliferation and dendritic cell maturation (68). Moreover, *MOV10*, in a model of porcine respiratory syndrome, blocked virus replication by affecting its nuclear import (69).

In conclusion, the TTV model seems to offer interesting explanations to the low COVID-19 percentages in Ph+ patients, showing that these subjects are probably less infected than healthy individuals and that TKIs seem to not favor viral replication.

On the other hand, the results from the second part of our study could help to reduce the doubt that TKIs might damage the immunological control of infection. Indeed, we observed the upregulation of some “pro-immune” genes, such as *CCL5, CD28*, and *IFNg*, and a reduced expression of some “anti-immune” genes, such as *ARG-1* and *FUT4*. We measured the expression of more than 700 genes involved in inflammation and immunity after 6 months of treatment with imatinib, a very long time with respect to that of a viral infection. This might not be a limitation of the study, because the absence of immunological damage after a medium-length treatment might further sustain the idea that TKIs would be not detrimental in shorter time intervals.

Certainly, we are aware that this study has some limitations, especially the low number of patients enrolled and the fact that the first time point of observation was, according to clinical practice, after 3 months of therapy. However, we think that our study might present some valid findings that could help to explain why very few Ph+ patients have been spared by SARS-CoV-2 or did not present severe forms of COVID-19. Obviously, we have to still wait for results of the ongoing large clinical trials before definitively establishing a possible effective role of TKIs in the coronavirus pandemic.

## Data Availability Statement

The original contributions presented in the study are included in the article/Supplementary Material, further inquiries can be directed to the corresponding author/s.

## Ethics Statement

Ethical approval was not provided for this study on human participants because at the first access to the Hematology Division, Pisa, all patients signed an informed consent approved by the Ethical Committee of the AOUP where they declared to donate leftover samples for further scientific non-profit purposes and for authorized researchers to use the clinical data. The patients/participants provided their written informed consent to participate in this study.

## Author Contributions

SG, MP, CBar, CBal, LB, and AD designed the study. CBal, FR, SB, and SM enrolled and managed patients. FG, SG, and EC conducted experiments and analyses. SG and FM wrote the first draft of the manuscript and prepared figures and tables. All authors reviewed the draft before submission.

## Conflict of Interest

SG, MP, and CBar participated to advisory boards organized by Novartis and were speakers during meeting supported by Novartis. The remaining authors declare that the research was conducted in the absence of any commercial or financial relationships that could be construed as a potential conflict of interest.
